# Comparison of SPH and CEL methods for simulating the soil cutting process of a biomimetic digging shovel

**DOI:** 10.1371/journal.pone.0322861

**Published:** 2025-05-22

**Authors:** Yunning Dong, Liangfei Fang, Kuan Qin, Shoujun Sun, Yue Gong

**Affiliations:** School of Engineering, Anhui Agricultural University, Hefei, Anhui, China; King Mongkut’s University of Technology North Bangkok, THAILAND

## Abstract

Changes in the resistance of tillage tools during cultivation are often investigated through field tests to reduce carbon emissions from agricultural machinery during tillage. Tillage tools are optimized using the test results to minimize energy loss. However, field tests typically face challenges such as high experimental costs, soil and climate limitations, and operational complexity. Numerical simulation, as a computer-aided research method, offers several advantages, including ease of use, cost-effectiveness, and resource conservation. This study employed the smoothed particle hydrodynamics (SPH) method and the coupled Eulerian-Lagrangian (CEL) method to examine the interaction between a biomimetic digging shovel and soil. In parallel, soil bin experiments were conducted, and the simulation results were compared to experimental data. The findings revealed that the simulated changes in cutting resistance were consistent with the experimental results, confirming the reliability of both models. Simulation results indicated that the CEL model required 6 h and 35 min to compute, while the SPH model required 7 h and 18 min. The relative error between the CEL model and the soil bin experiment was 8.81%, while that between the SPH model and the experiment was 13.76%. These results highlight the superior computational efficiency and higher computational accuracy of the CEL model. The validated CEL model was subsequently used to simulate the interactions between the digging shovel and soil under varying conditions.

## Introduction

Anhui is a major production area for rhizome-based Chinese medicinal crops, including Peucedanum praeruptorum, Paeonia lactiflora, and Pseudostellaria heterophylla. Although the mechanization of rhizome harvesting has become increasingly prevalent, the digging shovel—a key component of rhizome harvesters—remains a significant source of energy consumption. Excessive energy use in agricultural machinery leads to resource wastage and high exhaust emissions, which contribute to environmental pollution. Thus, optimizing the design of digging shovels for rhizome medicinal crops is crucial to reducing the resistance encountered during soil cutting and minimizing energy expenditure. With stringent carbon emission regulations in place, reducing the energy loss associated with digging shovels offers both economic and environmental benefits. Several studies have explored approaches to lowering the resistance of digging shovels from both macro and micro perspectives. Methods examined include modifying surface material properties[1], incorporating vibratory mechanisms [2], and refining shovel profile designs [3].

However, reducing shovel resistance through environmental modifications or additional equipment often requires more energy or higher mechanical stability, posing limitations in practical applications. Material enhancements for digging shovels, while promising, tend to be costly and may offer limited economic advantages. Given the suboptimal performance of standard plane shovels in resistance reduction, researchers have increasingly focused on modifying the shovel profile as an effective strategy to reduce resistance during tillage.

The biomimetic approach has emerged as a particularly effective design methodology for shovel profile improvement. By emulating natural forms, biomimetic digging shovels can significantly reduce cutting resistance without requiring heightened mechanical stability, thereby conserving energy. For example, researchers have developed biomimetic designs by modeling shovel blades after the mole’s toe [4], the boar’s snout [5], and the sandfish lizard’s head [6]. Experimental results consistently indicate that these biomimetic shovels demonstrate superior resistance reduction compared to standard plane shovels, underscoring the effectiveness of biomimicry in shovel design optimization. Consequently, the biomimetic approach is regarded as a viable and efficient strategy for enhancing shovel structure, with the ultimate goal of reducing cutting resistance during tillage operations.

Researchers have evaluated the resistance reduction performance of digging shovels through theoretical analysis, experimental methods, and simulation techniques to investigate the tillage process of these shovels [7]. However, in practical engineering applications, analytical methods are typically unsuitable for biomimetic digging shovels with complex structures due to computational limitations. Solutions derived from analytical models are often imprecise for these intricate designs [8,9]. Experimental methods yield more accurate results but require substantial labor, materials, and time[10–12]. While simulation methods offer a more efficient alternative by numerically modeling the soil tillage process, discrepancies between simulated and actual data are common, though these errors can be minimized with improved simulation techniques. Therefore, numerical simulation methods have become increasingly popular for assessing digging shovel performance.

Currently, three primary numerical simulation methods are commonly used. The first is the discrete element method (DEM)[7,13–15], which effectively simulates soil tillage processes but lacks direct stress-strain modeling capabilities, requiring complex calibration for soil mechanics parameters [16,17]. The second is computational fluid dynamics (CFD)[18–20], which uses Eulerian descriptions to prevent element destruction. However, this approach is unsuitable when the material boundary deforms, as it employs a fixed mesh in space[21]. The third is the finite element method (FEM)[22–24], wherein the conventional Lagrangian approach describes soil material, with nodes fixed within the material that deform with it. This approach leads to severe mesh distortion during large soil deformations, which can compromise accuracy or even halt computation [25,26].

To address mesh distortion arising from significant soil deformation, researchers have developed methods that improve upon these three approaches, including the arbitrary Lagrangian-Eulerian (ALE) method[21], the smoothed particle hydrodynamics (SPH) method[27], and the coupled Eulerian-Lagrangian (CEL) method [28]. These methods partially resolve the issue of severe mesh distortion. The ALE method, an arbitrary Lagrangian-Eulerian approach, is primarily used for two-dimensional cutting simulations. The SPH method, a meshless Lagrangian technique[27], discretizes the continuum into particles and was initially applied to astrophysical problems; it has since been adapted for applications such as fluid flow, elastoplastic flow, brittle material failure, and soil cutting processes [29,30]. The CEL method, a coupled fluid-solid approach, uses the Eulerian method for fluids and the Lagrangian method for solids, combining the strengths of both to address mesh distortion issues caused by large soil deformations[28]. Compared with the ALE method, CEL and SPH are better suited for three-dimensional soil-tool interaction simulations.

Zhang et al. employed the SPH method to investigate the soil-cutting process of a typical rotating blade[31]. Fang et al. developed a coupled numerical model based on the SPH method, incorporating the interaction between deformable tools and soil[32]. Hu et al. established an SPH-based soil-tool interaction model using an elastoplastic constitutive and validated its feasibility and precision[33]. Yang et al. constructed a simulation model of a spiral cutter-soil system using the SPH method to investigate the working process and forces acting on a spiral cutter[34]. Li et al. conducted soil-cutting simulations with SPH modeling to optimize rotary blade design and reduce energy consumption during tillage operations[35]. R. Skirkus et al.modeled the contact between soil and cultivator tips based on the CEL method[36]. Hu, Desheng et al. used the CEL method to simulate the three-dimensional drilling model[37]. The simulation results of the drilling force obtained by the CEL method are compared with the experimental results. Pan Gao et al. set up a finite element method model of anchor penetration with the CEL method[38].They analysed the soil deformation and the effects of strain and strain rate with the CEL model,and validated the model against the test results. Gao, Lei et al. simulated the large deformation of the soil around the pile during the penetration of the snowflake pile based on the CEL method[39].

Despite these advancements, no studies in the existing literature have reported soil-cutting simulations for wide-toothed tillage tools, such as digging shovels, using the CEL method. Therefore, this study aims to assess the accuracy and effectiveness of the CEL and SPH method in modeling soil-wide-toothed tillage tool interactions. Furthermore, the validated soil-tool model is employed to examine the resistance encountered by a biomimetic digging shovel during cultivation, facilitating shape optimization and reducing energy loss. The specific objectives of this study are to (1) simulate biomimetic digging shovel-soil interactions using the CEL and SPH methods in the finite element software ABAQUS, (2) validate the reliability of these methods by comparing simulation results with soil bin experimental data, and (3) investigate the effects of cutting depth, cutting speed, and shovel curvature on soil cutting resistance using the validated simulation models.

## Materials and methods

### Overview of SPH and CEL methods

#### SPH method.

The SPH method is based on the principles of kernel approximation and particle approximation[40]. In kernel approximation theory, the kernel function *W* is introduced to describe the interaction between particles. An arbitrary continuous function *f*(*r*) and its derivative ∇*f*(*r*) can be expressed as follows:

$$f\left(r\right)=\int_{𝛺}f\left(r^{\prime }\right)W\left(r-r^{\prime },h\right)dr^{\prime }$$ (1)

$$\nabla f\left(r\right)=\int_{𝛺}f\left(r^{\prime }\right)\nabla_{r}W\left(r-r^{\prime },h\right)dr$$ (2)

where 𝛺 represents the problem domain, *h* denotes the smoothing length defining the domain of support of the kernel function *W*, ∇_*r*_represents the gradient of the relative position *r*.

In the standard SPH framework, the function value of particle *i* and its derivative are obtained by interpolating values from other particles within the support domain, expressed as follows:

$$f\left(r_{i}\right)=\sum_{j=1}^{N}f\left(r_{j}\right)W\left(r_{i}-r_{j},h\right)\frac{m_{j}}{\rho_{j}}$$ (3)

$$\nabla f\left(r_{i}\right)=\sum_{j=1}^{N}f\left(r_{j}\right)\nabla_{i}W\left(r_{i}-r_{j},h\right)\frac{m_{j}}{\rho_{j}}$$ (4)

where subscripts *i* and *j* denote particle indices, *N* denotes the total number of particles *i* in the support domain of particle, *m*_*j*_ and ρ_j_are the mass and density of particle *j*, respectively.

The performance of the SPH model is highly dependent on the choice of the kernel function, which must meet conditions such as compact support, regularity, and compactness. The kernel function must decay as the distance between interacting particles increases, approximate the Dirac *δ* function, and exhibit a large central peak to enhance approximation accuracy. The Dirac *δ* function and the cubic spline kernel function are shown in (Eq 5) and (Eq 6).

$$\delta \left(r-r^{\prime }\right)=\left\{ \begin{array}{cc} 1, & r=r^{\prime }\\ 0, & r\neq r^{\prime } \end{array}\right.$$ (5)

$$W(r-r^{\prime },h)=\alpha_{d}\{\begin{array}{ll} \frac{2}{3}-R^{2}+\frac{1}{2}R^{3}, & 0\leq R<1\\ \frac{1}{6}{\left(2-R\right)}^{3}, & 1\leq R<2\\ 0, & R\geq 2 \end{array}$$ (6)

where *R* = *r*/*h* is the relative distance between the two particles at point *r* and $r^{\prime }$ . In one, two, and three dimensions, $\alpha_{d}=1/h$,$15/7\pi h^{2}$ and $3/2\pi h^{3}$ , respectively.

#### CEL method.

In ABAQUS, the CEL method uses the dynamic explicit algorithm with a central difference method for Lagrangian analysis[41]. For problems involving large deformations, the central difference method’s critical time step diminishes with decreasing element characteristic lengths. The variable time step is applied in the following procedure:

Given the initial velocity ${\dot{u}}_{0}$ and displacement *u*_0_ of the node at the moment *t* = 0, the initial acceleration ${\ddot{u}}_{0}$ is calculated using the equation of motion (7):

$${\ddot{u}}_{0}=M^{-1}f_{0}$$ (7)

where *M* denotes the mass matrix;*f*_0_ denotes the total nodal force at the moment *t* = 0.

The initial velocity ${\dot{u}}_{-1/2}$ at *t* = −1/2 is calculated as follows:

$${\dot{u}}_{-1/2}={\dot{u}}_{0}-\frac{1}{2}\Delta t_{-1}{\ddot{u}}_{0}$$ (8)

where $\Delta t_{-1}$ represents the incremental time step from *t* = −1 to *t* = 0, that is $\Delta t_{-1}=t_{0}-t_{-1}$.

The following parameters are calculated for each time increment step, starting from the 0th time increment step (*n* = 0):

1. velocity ${\dot{u}}_{n+1/2}$ at the moment *t* = *n* + 1/2:
  $${\dot{u}}_{n+1/2}={\dot{u}}_{n-1/2}+\frac{1}{2}(\Delta t_{n-1}+\Delta t_{n}){\ddot{u}}_{n}$$ (9)
2. Displacement *u*_*n* + 1_ at the moment *t* = *n* + 1:
  $$u_{n+1}=u_{n}+\Delta t_{n}{\dot{u}}_{n+1/2}$$ (10)
3. Element strain $\varepsilon_{n+1}$ and strain rate $\varepsilon_{n+1}$ are calculated using a geometric equation.
4. Element strain increment $d\varepsilon_{n+1}$:
  $$d\sigma_{n+1}=D^{e,p}d\varepsilon_{n+1}$$ (11)
5. Element stress increment $d\sigma_{n+1}$:
  $$d\sigma_{n+1}=D^{e,p}d\varepsilon_{n+1}$$ (12)
6. where *D*^*e*,*p*^ represents the elastic-plastic matrix.
7. Stress *σ*_*n*+1_ at the moment:*t* = *n* + 1
  $$\sigma_{n+1}=\sigma_{n}+d\sigma_{n+1}$$ (13)
8. Update the total nodal force *f*_*n* + 1_ at the moment *t* = *n* + 1;
9. Acceleration ${\ddot{u}}_{n+1}$ at the moment *t* = *n* + 1 is calculated using the equation of motion (14) at the moment *t* = *n* + 1:
  $${\ddot{u}}_{n+1}=M^{-1}f_{n+1}$$ (14)
10. *n* = *n* + 1 for the next time increment step.

**Fig 1:**
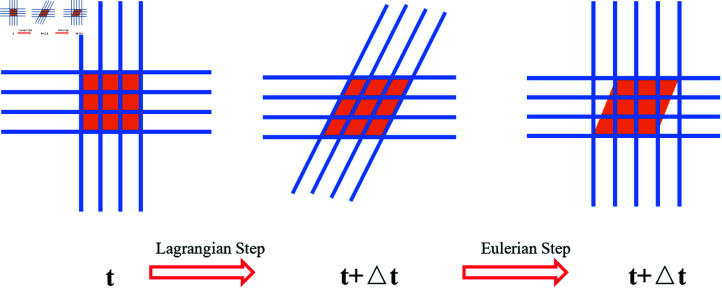
Diagram illustrating the operator split implementation.

This incremental algorithm is based on operator splitting of control equations, where a Lagrangian step is followed by an Eulerian step. The implementation of the operator split is illustrated in Fig 1

### Constitutive model of soil

ABAQUS finite element software provides an extensive library of material models suitable for simulating a wide range of engineering materials. Soil, characterized by nonlinear and complex mechanical behavior, can be represented by three constitutive models: the Mohr-Coulomb model [42], the Drucker-Prager model [43], and the Cam-Clay model [44].

He et al. [45]applied the linear form of the extended Drucker-Prager model in ABAQUS/Explicit to simulate soil, demonstrating its suitability and accuracy for soil materials. Given that the soil in this study is sandy loam, this model was also selected to simulate its stress-strain behavior. The model is defined as follows:

F=t−ptanβ−d (15)

where *F* represents the yield function, *t* denotes the deviatoric stress parameter using (Eq 16),*p* denotes the equivalent pressure stress using (Eq 17), and *β* denotes the inclination of the yield surface in the *p*–*t* stress space, which is a function of the friction angle *φ* of the material calculated using (Eq 20). The parameter *d* denotes the intercept of the yield surface on the t-axis in the *p*–*t* stress space, representing the cohesion of the material.

$$t=\frac{1}{2}q\left[1+\frac{1}{k}-\left(1-\frac{1}{k}\right){\left(\frac{r}{q}\right)}^{3}\right]$$ (16)

$$P=\frac{1}{3}\left(\sigma_{1}+\sigma_{2}+\sigma_{3}\right)$$ (17)

$$r=-q=-(\sigma_{1}-\sigma_{3})$$ (18)

$$k=\frac{3-\sin\phi }{3+\sin\phi }$$ (19)

$$\tan\beta =\frac{6\sin\phi }{3-\sin\phi }$$ (20)

where *σ*_1_,*σ*_2_ and *σ*_3_ represent the compressive stress in a triaxial test *t*; *r* represents the third invariant of deviatoric stress; and *q* represents the mises equivalent stress. The parameter *k* is defined as the ratio of the yield stress in triaxial tension to the yield stress in triaxial compression, controlling the influence of the intermediate principal stress on the yield surface. To ensure convexity of the yield surface *k*, must satisfy the condition 0.778 ≤ *k* ≤ 1.

The sandy loam soil used in this study was obtained from the soil bin in the Agricultural Machinery Laboratory at Anhui Agricultural University, Anhui Province, China. The soil’s Young’s modulus *E* and Poisson’s ratio *μ* were determined through triaxial compression tests conducted under a confining pressure of 100 kPa, which approximates atmospheric pressure[46]. The internal friction angle *ϕ* for the Mohr-Coulomb model was calculated using data from triaxial compression tests performed at confining pressures of 50 kPa, 100 kPa, and 150 kPa. The flow stress ratio *k* and internal friction angle *β* for the extended Drucker-Prager model were then derived from (Eq 19) and (Eq 20), respectively. Other parameters were set to default values in ABAQUS software (Dassault Systèmes Simulia Corp., Providence, RI, USA). The simulation parameters for both the soil and the digging shovel used in this study are presented in Table 1.

**Table 1:** Simulation parameters

Parameters	Soil	biomimetic digging shovel	Data sources
Density/(*g*/*cm*^3^)	1.76	7.98	Soil measurement values
moisture content/%	20.25	-	Soil measurement values
Poisson’s ratio	0.432	0.3	Soil measurement values
Young’s modulus/*MPa*	6.17	2.1×10^5^	Soil measurement values
angle of internal friction/°	14.33	-	Soil measurement values
cohesion/*kPa*	13.98	-	Soil measurement values
Soil-shovel friction coefficient	0.5	0.5	Soil measurement values
Acceleration due to gravity/*ms*^−2^	9.8	9.8	-

## Simulation of soil-shovel interaction

### Biomimetic digging shovel

**Fig 2:**
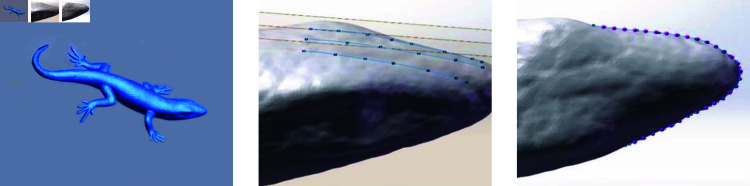
Extraction of contour lines from the sandfish lizard’s appearance.

Biomimetic digging shovels have been proposed as a means to reduce cutting resistance during tillage. Several studies have investigated various designs of biomimetic digging shovels using numerical simulations and physical experiments. This study examines a biomimetic digging shovel inspired by the sandfish lizard, specifically focusing on the structure of its head, which exhibits notable ground-penetration and resistance-reduction capabilities. The shovel blade structure is designed based on curve equations fitted to the side and top profiles of the sandfish lizard’s head. The first step of the process involves scanning and modeling the sandfish lizard’s head, after which the model is imported into SolidWorks. Using the “Art Spline" command, contour lines of the head’s side and top views are drawn. Two equidistant reference surfaces are created on the side view, along with a reference surface on the top view. Uniformly spaced points are marked along the contour lines, and the coordinates are extracted and imported into Origin to generate the fitted curves. The contour line extraction process of the sandfish lizard’s head is illustrated in Fig 2.

**Fig 3:**
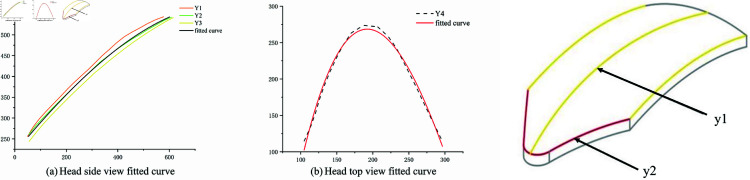
Fitted curves of the sandfish lizard’s head.

The three ridges in the lateral profile of the sandfish lizard’s head are fitted into a single curve (Fig 3), denoted as (y1), using a polynomial function. Similarly, a polynomial function is applied to the top profile, resulting in a fitted curve (y2). These equations are expressed as follows:

$$\begin{array}{c} y_{1}=3.56721\times 10^{-5}x^{3}-0.03913x^{2}+11.09368x-671.46723\\ \left(R^{2}=0.988\right) \end{array}$$ (21)

$$\begin{array}{c} y_{2}=-1.60698\times 10^{-7}x^{3}-2.54927\times 10^{-4}x^{2}+0.75447x+216.42867\\ \left(R^{2}=0.989\right) \end{array}$$ (22)

### Finite element modeling of biomimetic digging shovels

**Fig 4:**
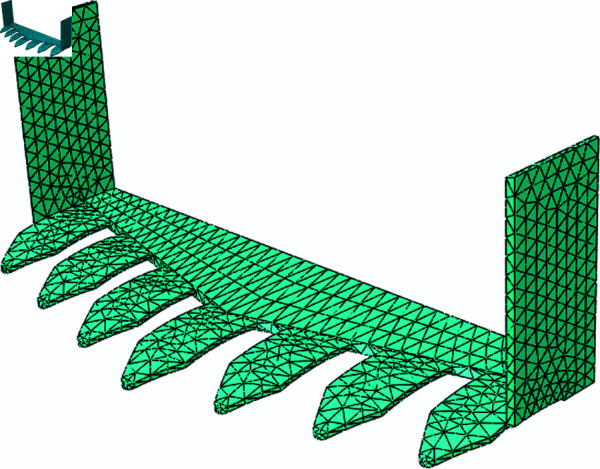
Geometry and mesh distribution of the biomimetic digging shovel.

The study focuses on a biomimetic digging shovel designed for harvesting rhizome-based traditional Chinese medicinal plants. This shovel, inspired by the wing structure of the sandfish lizard’s head, has a blade width of 400 mm. The 3D model of the shovel was created in SolidWorks, converted to STEP format, and imported into ABAQUS to develop a finite element model. Due to the complex geometry of the shovel, a free mesh distribution was applied in the ABAQUS Mesh module, with an element size of 10 mm and element type set to C3D10M. Material properties were assigned in the Property module, with 65Mn steel selected for the shovel, characterized by a density of 7.98*g*/*cm*^3^, a Poisson’s ratio of 0.3, and a Young’s modulus of 2.1×10^5^*MPa*. The geometry and mesh distribution of the digging shovel are presented in Fig 4.

### SPH soil-shovel interaction model

**Fig 5:**
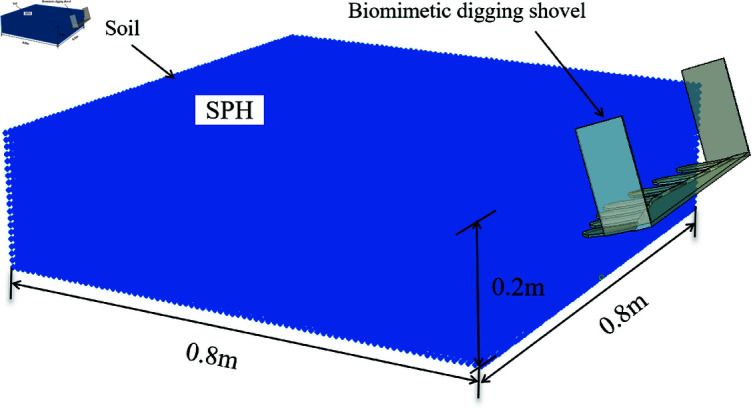
SPH soil-shovel interaction model.

In ABAQUS, generating SPH particles requires first establishing a finite element mesh and then converting it into SPH particles at the initial step. A file containing the SPH particle model is then generated and imported into the Part module. The SPH soil model used in this study has dimensions of 800 × 800 × 200 mm, with hexahedral elements of 5 mm each, and the element type set to C3D8R. The final SPH particle model is illustrated Fig 5.

### CEL soil-shovel interaction model

**Fig 6:**
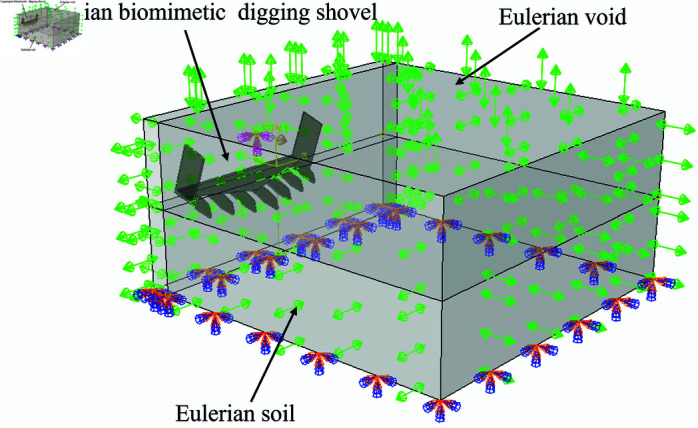
CEL soil-tool interaction model.

The CEL soil model was constructed with dimensions consistent with those of the SPH model. In the CEL model, the Eulerian domain is divided into two regions: one containing soil material (the Eulerian soil region) and an adjacent empty region to accommodate soil deformation and movement. During the soil-tool interaction simulation, the Eulerian soil undergoes deformation and displacement. If the soil moves beyond the Eulerian domain, it is excluded from the simulation. To prevent this issue, the void region is designed with sufficient dimensions to fully accommodate soil movement and deformation. The Eulerian soil elements are defined using the EC3D8R element type with a 5 mm element size. The generated cubic elements are uniformly distributed throughout the Eulerian domain, as shown in Fig 6.

### Boundary condition settings

The assembled model includes both the soil and the biomimetic digging shovel, with a digging depth set to 100 mm and an entry angle of 20°. A general contact is defined between the shovel and soil, and gravity is applied across the entire model. The biomimetic digging shovel is simulated in the Lagrangian region and constrained as a rigid body with six degrees of freedom. The tool’s uniform velocity is set to 600 mm/s along the x-axis. The rigid body constraint prevents deformation of the shovel during the cutting process, enhancing computational efficiency and allowing direct application of boundary conditions to the rigid body’s reference points. Fixed constraints are applied to the bottom surface of both the SPH and CEL soil models, while the remaining surfaces are free. A dynamic explicit analysis step is defined with a solution time of 1.65 s.

### Parameter study of the biomimetic digging shovel

**Fig 7:**
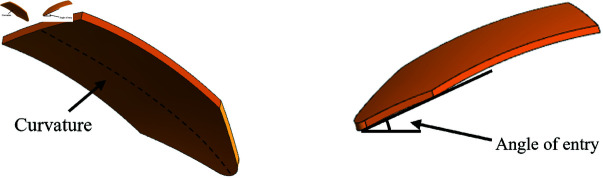
Curvature and angle of entry for the biomimetic digging shovel.

Further modeling was conducted using the CEL method to optimize the biomimetic digging shovel design. The study investigated the optimal shovel parameters by adjusting working conditions, including depth, speed, entry angle, and blade curvature (Fig 7). Forward speed was controlled between 0.6 and 1.0 m/s to assess resistance variation with speed across different depths. The entry angle was varied from 15° to 30° to evaluate resistance changes with angle at various depths. Blade curvature was adjusted between 2.2 and 3.2*m*^−1^ to simulate resistance variation relative to shovel curvature at different depths.

### Experimental setup for soil cutting

**Fig 8:**
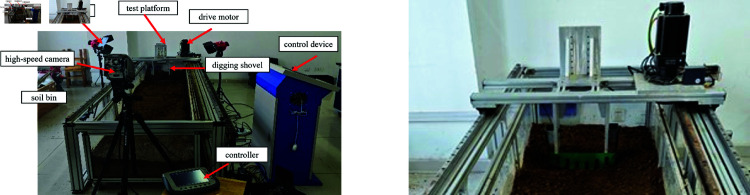
Experimental equipment and devices.

The experimental apparatus utilized in this study is a digital soil bin test platform, comprising the main frame, soil bin box, motion mechanism, and control collection mechanism, among other components. As shown in Fig 8, the soil bin box measures 3.8 m in length and 0.9 m in width. Based on simulation parameters, the biomimetic digging shovel was set to a depth of 100 mm, with a digging speed of 600 mm/s and a total digging time of 1.3 s. The entry angle into the soil was fixed at 20°. Multiple sets of soil-shovel interaction experiments were conducted, and average values were recorded for analysis.

## Results and discussion

### Cutting process and cutting resistance analysis

**Fig 9:**
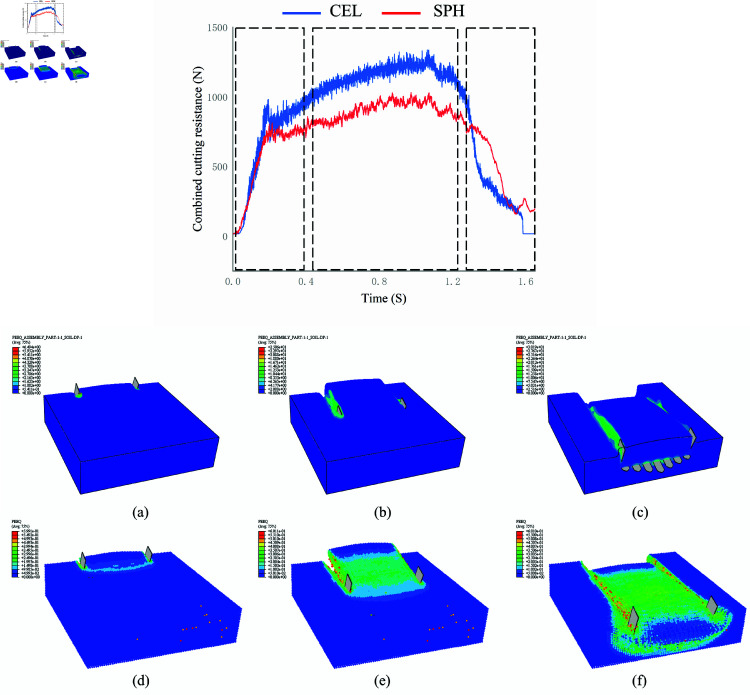
Three main stages of soil cutting.

Fig 9 illustrates the soil cutting process simulated using both the CEL and SPH methods, highlighting the degree of soil deformation and displacement at three key stages: the initial contact between the digging shovel and the soil, the moment when the digging shovel fully penetrates the soil, and the point when the digging shovel separates from the soil. Upon initial contact, the soil experiences failure due to shear forces from the shovel and compression between the soil particles. As the contact area between the digging shovel and the soil increases, the stress within the soil also increases.

When the biomimetic digging shovel begins to separate from the soil, the soil continues to move due to inertia, and compression between the soil particles persists. Consequently, the biomimetic digging shovel continues to cut through the soil. The soil cutting processes simulated using both methods are stable and reliable, accurately reflecting the interactions between the soil particles. The simulation results are consistent with the soil bin experiments, which indirectly validate the reliability of the simulation model.

The force exerted on the digging shovel corresponds to the reaction force encountered during the soil cutting process. Therefore, the reaction forces in both horizontal and vertical directions were added to the historical output variables of ABAQUS. Fig 9 shows the curves of the combined reaction force obtained from the SPH and CEL models. When the digging shovel is not in contact with the soil, the cutting resistance is zero. As the contact area between the digging shovel and the soil increases, the cutting resistance rises. At 0.8 s, the cutting resistance reaches its peak value and fluctuates. When the digging shovel starts to leave the soil at 1.2 s, the cutting resistance gradually decreases. By 1.65 s, when the digging shovel has completely separated from the soil, the cutting resistance returns to zero. The digging shovel fully penetrates the soil at 0.4 s and begins to separate at 1.2 s. The combined cutting resistance data from the CEL and SPH models between 0.4 s and 1.2 s were extracted for analysis. The calculated average cutting resistances for the SPH and CEL models were 903.82 N and 1153.40 N, respectively.

### Soil cutting shapes analysis

**Fig 10:**
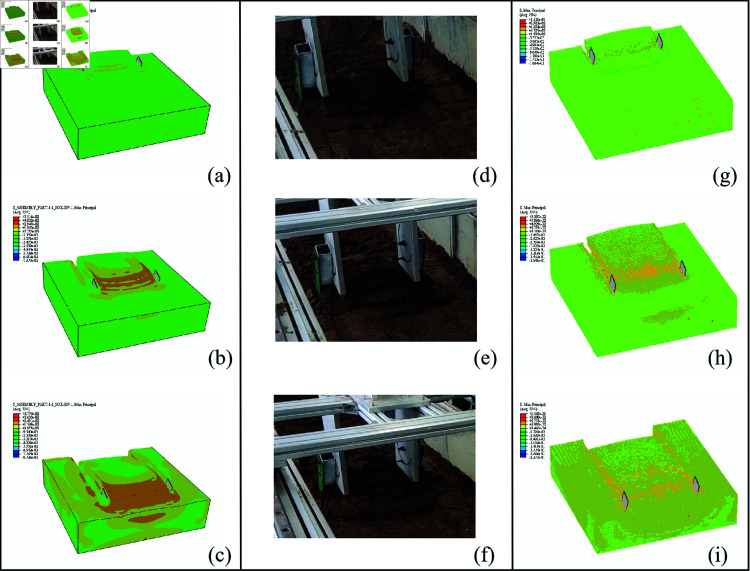
Comparison of soil destruction between the simulation model and soil bin experiment at different time intervals. CEL model: (a) t = 0.2 s, (b) t = 0.8 s, (c) t = 1.4 s; Soil bin experiment: (d) t = 0.2 s, (e) t = 0.8 s, (f) t = 1.4 s; SPH model: (g) t = 0.2 s, (h) t = 0.8 s, (i) t = 1.4 s.

The soil cutting process of a digging shovel can be characterized as the interaction between an inclined, wide tillage tool and the soil, where the tool cuts in a straight line at a low speed. A wide tillage tool is defined as one in which the ratio of tillage depth to tool width is less than 0.5[47]. The primary feature of wide-tool soil cutting is the repeated failure and fracturing of the soil because of continuous compression, shearing, and tension, resulting in the formation of numerous small soil blocks [48]. This study examines the interaction between the soil and the digging shovel through cutting simulations and compares the results with actual soil bin test data to validate the simulation model, as shown in Fig 10. According to Fig 10, the SPH-based soil-cutting simulation does not clearly depict the soil separation surface. By contrast, the CEL soil-tool interaction model predicts soil-cutting patterns that are more similar to those observed in the soil bin experiments.

**Fig 11:**
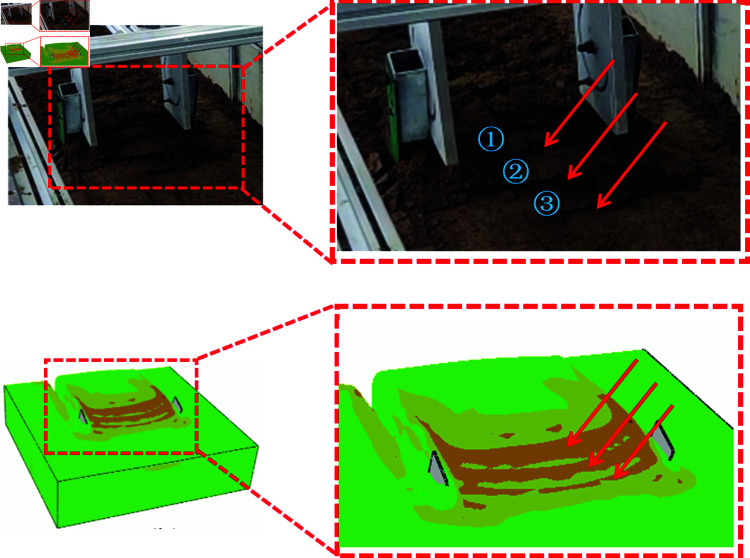
Cracks and small soil blocks formed during the soil cutting process.

At the onset of cutting, the digging shovel contacts the soil and applies a cutting force. This force causes the soil particles to move, which in turn leads to changes in the internal structure and stress distribution of the soil. As the digging shovel progresses further into the soil, internal shear stresses are continuously generated, propagating along the tangent direction. This propagation causes further displacement of the soil particles and results in backward extrusion until failure cracks appear on the soil block on the shovel surface. As the digging shovel advances, the cracks propagate, and the soil block on the shovel surface breaks into smaller fragments that move upward and backward along the surface of the digging shovel. Once the cutting force stabilizes, the failure pattern of the soil on the shovel surface also stabilizes, with regular formation of cracks and continuous production of small soil blocks. As illustrated in Fig 11, the digging shovel continuously cuts through the soil, repeatedly forming small soil blocks, similar to those observed in stages ①-③.

The cracks observed in the soil blocks on the shovel face are a direct result of the internal stress of the soil exceeding the material strength limit during the cutting process. The simulation allows for observation of the stress transmission and distribution behavior within the soil during cutting. In comparison to the SPH method, the simulation results from the CEL method more clearly demonstrate that the distribution and position of the maximum stress values within the soil blocks on the shovel surface correspond to the locations of the cracks observed in the soil bin tests, as shown in Fig 11.

### Model validation

**Fig 12:**
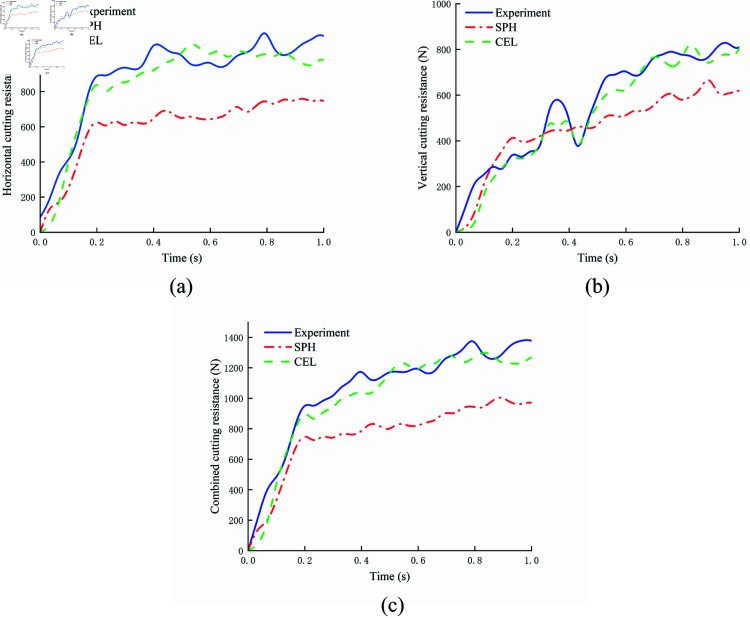
Comparison of cutting resistance data from CEL and SPH simulation models and soil bin experiments. (a) horizontal; (b) vertical; (c) combined cutting resistance.

**Fig 13:**
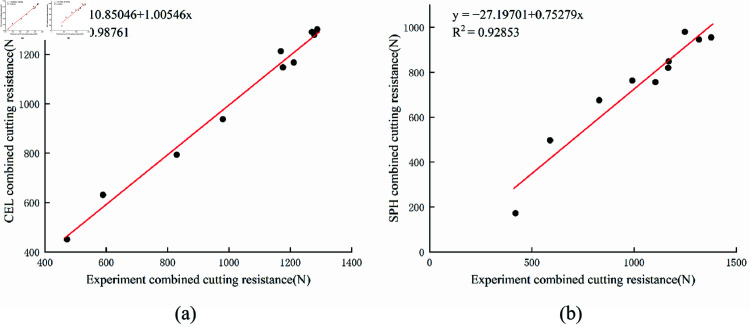
The linear regression plots for the estimated against measured. (a) CEL; (b) SPH.

The soil cutting process can be simulated using several existing methods, and discrepancies between simulation and experimental results are inevitable. Therefore, the simulation model must be validated to ensure the accuracy and reliability of the chosen methods. This study compares the soil cutting simulations using the CEL and SPH methods with soil bin experiment data to assess the reliability of both approaches. Fig 12 presents the curves of vertical and horizontal cutting resistance of the biomimetic digging shovel over time. The parameters for both the simulation and soil bin experiment are as follows: cutting speed of 0.6 m/s, cutting depth of 100 mm, and an entry angle of 20°. The comparison reveals that the cutting resistance curves from both simulation methods closely align with the experimental data, thereby confirming the reliability of the models. Fig 13 presents the linear regression plots of combined cutting resistance for the soil bin experiment and the simulations. The CEL model exhibits the highest regression accuracy for combined cutting resistance.

At 0.1 s, when the biomimetic digging shovel contacts the soil, the cutting resistance begins to rise as the soil around the shovel is displaced. The horizontal and vertical resistance values increase in response to the accumulation of disturbed soil around the shovel. Subsequently, due to spatial constraints, the accumulated soil volume reaches an equilibrium state and fluctuates around this value, causing the cutting resistance to stabilize. The average resistance from the CEL simulation model is 1139.50 N, while the average resistance from the SPH simulation model is 903.11 N. The average resistance from the soil bin experiment is 1047.26 N. Discrepancies between the simulation models and experimental results arise due to the complexity of actual soil properties, which are influenced by processing variations and environmental factors. Simulation software cannot fully replicate the physical characteristics, constraints, and loading conditions of real soil. The CEL simulation model deviates from the soil bin experiment by 8.81%, whereas the SPH simulation model exhibits a relative error of 13.76%, indicating the superior accuracy of the CEL method.

In terms of computational accuracy, the CEL model demonstrates superior performance. Furthermore, in terms of computational volume and efficiency, the SPH model required 7 h and 18 min, whereas the CEL model completed the simulation in just 6 h and 35 min. To minimize simulation errors, researchers typically refine the mesh size or increase the number of particles, often leading to longer computational times and necessitating advanced hardware. However, adopting the CEL method effectively reduces simulation time and dependence on high-cost hardware and accelerates the execution of multiple comparative experiments. Shorter simulation times facilitate faster feedback, facilitating the timely identification and resolution of problems and ultimately enhancing research progress. The CEL model thus offers higher efficiency and a smaller computational volume. In conclusion, the CEL method provides a more accurate and efficient approach for simulating the soil cutting process compared to the SPH method.

Both the CEL and SPH methods are effective for simulating soil cutting by tillage tools, yielding satisfactory results. The cutting process can be regarded as a fluid-solid coupling simulation, where the soil can behaves as a fluid and the tool as a solid. The CEL model produces results more consistent with experimental data, likely because it employs a fluid-solid coupling framework. Eulerian elements are used to model fluids or quasi-fluids, while Lagrangian elements represent solids and other non-fluids. In an Eulerian grid, the grid remains stationary, while the material flows within it. At each incremental step, the material distribution within each element is calculated, describing fluid deformation through its distribution. Therefore, Eulerian material boundaries are more suitable for large deformations than Lagrangian material boundaries. Consequently, CEL is advantageous in solving problems involving dynamic tool-soil interactions and extended computational cycles. Furthermore, this study reveals that the CEL method demonstrates a higher computational efficiency than the SPH method when the number of grid elements is small, the computational speed of both methods is basically the same. However, as the number of grid elements increases, the CEL method demonstrates higher computational efficiency than that of the SPH method, highlighting its potential for simulating tillage tool-soil interactions. This study focuses on loam soil, and cutting simulations for other soil textures have not been conducted. Additionally, only one type of tillage tool was examined, presenting certain research limitations. Soil exhibits highly complex properties, and variations in physical parameters such as moisture content and compaction can influence both experimental and simulation results. Future research will extend cutting simulations to diverse soil types, including sandy soil and clay, under varying physical conditions to further explore the applicability of different simulation methods across various soil-cutting scenarios.

### Effects of cutting speed and depth on cutting resistance

**Fig 14:**
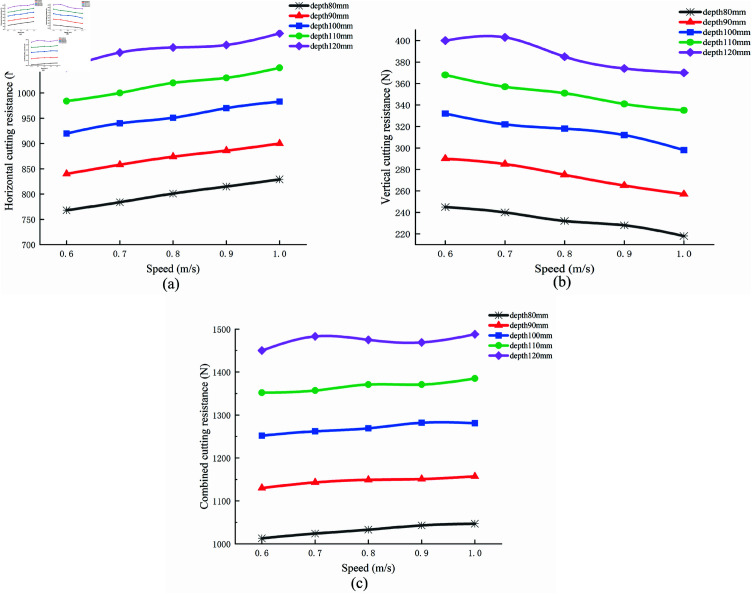
Average cutting resistance at different speeds and depths. (a) horizontal ; (b) vertical; (c) combined cutting resistance.

Fig 14 illustrates the average cutting resistance at various cutting speeds and depths. The cutting depths considered were 80 mm, 100 mm, and 120 mm, while the cutting speeds ranged from 0.6 m/s to 1.0 m/s in increments of 0.1 m/s. The results indicate an increasing trend in horizontal cutting resistance as the cutting speed increases. At a cutting depth of 80 mm, the shovel experiences the smallest resistance, while at a depth of 120 mm, the largest resistance is observed. This shows that cutting resistance increases with greater cutting depth. At the same depth, resistance increases with speed, although the rate of increase declines with increasing speed. The cutting depth of the biomimetic digging shovel exerts a more significant influence on horizontal cutting resistance than cutting speed. Specifically, horizontal cutting resistance is lowest at a cutting speed of 0.6 m/s and highest at 1.0 m/s, with resistance increasing steadily with speed. In contrast, vertical cutting resistance increases with depth, though it decreases slightly as speed increases.

### Effects of angle of entry on cutting resistance

**Fig 15:**
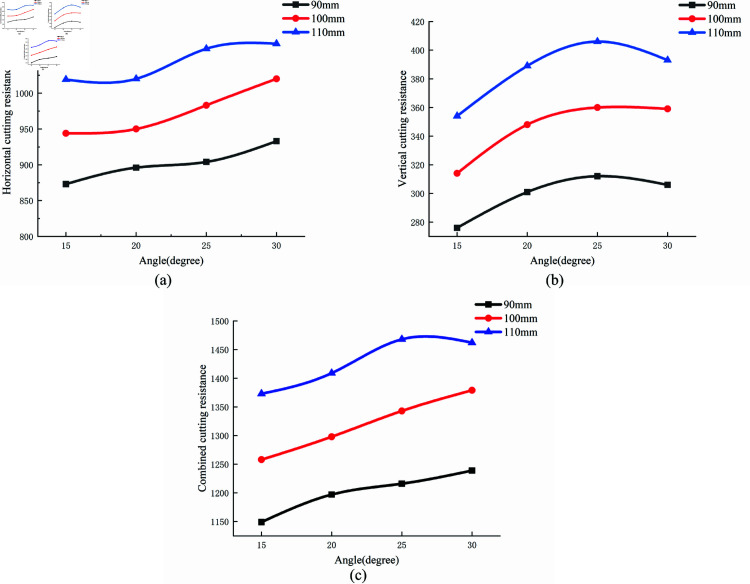
Average cutting resistance at different angles and depths. (a) horizontal; (b) vertical; (c) combined cutting resistance.

Fig 15 presents the average cutting resistance at various angles of entry (15°, 20°, 25°, and 30°) and cutting depths. As the angle of entry increases, both horizontal and vertical cutting resistance, as well as the combined cutting resistance, exhibit an increasing trend. Additionally, cutting resistance increases substantially as the cutting depth increases.

### Effects of curvature on cutting resistance

**Fig 16:**
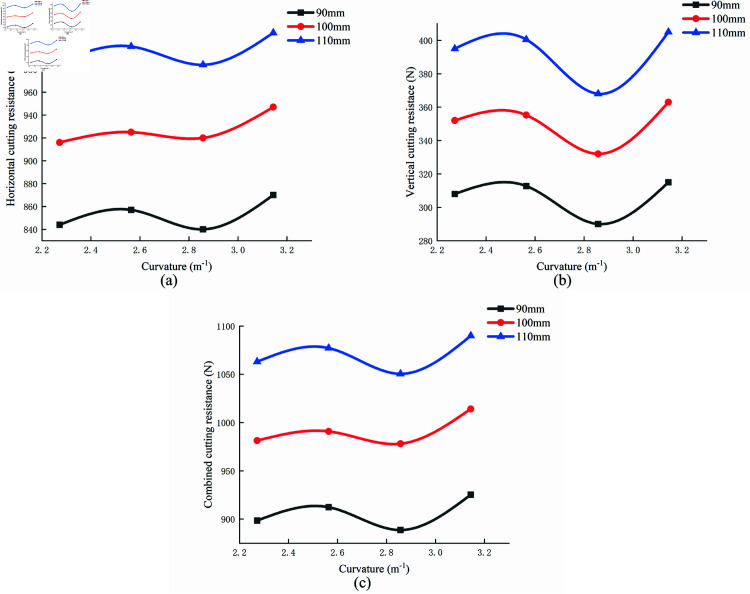
Average cutting resistance at different curvatures and depths. (a) horizontal; (b) vertical; (c) combined cutting resistance.

Fig 16 illustrates the variation in cutting resistance at different depths as the curvature of the digging shovel changes, with a cutting speed set at 0.6 m/s. The results demonstrate that horizontal, vertical, and combined cutting resistances are minimized when the curvature is 2.857. This curvature value, extracted and fitted from the sandfish lizard prototype, represents the optimal configuration, as it corresponds to the minimum cutting resistance. These findings further confirm that the biomimetic digging shovel exhibits enhanced performance in terms of resistance reduction.

## Conclusion

This study employed the CEL and SPH methods to simulate the interaction between soil and a biomimetic digging shovel during the cutting process. A corresponding soil bin experiment was conducted to validate the simulations, and the results were compared. The soil cutting resistance curves obtained from the simulations were found to be consistent with those from the soil bin tests. The average cutting resistance from the SPH simulation was 903.11 N, while the CEL simulation yielded an average resistance of 1139.50 N. The relative errors were 13.76% for the SPH method and 8.81% for the CEL method, demonstrating the greater reliability of the CEL approach. A comparison of the simulated soil cutting process using the CEL method with the results from the soil bin experiment showed that the failure patterns of the soil were similar, highlighting the advantage of the CEL method in simulating large deformation during soil cutting. The CEL model demonstrated superior computational accuracy, with smaller computational volume and higher efficiency compared to the SPH model. Overall, the CEL method proved to be a more accurate and efficient approach for simulating the soil cutting process, effectively conserving computational resources and facilitating the optimization of biomimetic shovel design.

The CEL method was also applied to analyze the soil failure process, providing deeper insights into failure mechanisms. This analysis can inform the optimization of tillage components in agricultural machinery, reducing cutting resistance and consequently lowering energy consumption. The development of optimized tillage components can significantly mitigate carbon emissions and environmental pollution associated with agricultural machinery operations.

In addition, the study investigated the effects of cutting depth, curvature, and speed on the cutting resistance of the biomimetic digging shovel. The results indicated that cutting depth had a more significant effect on resistance than cutting speed. The curvature of the biomimetic digging shovel also influenced resistance, with a curvature value of 2.857*m*^−1^ yielding the minimum resistance. These findings confirm the effectiveness of the biomimetic design in reducing cutting resistance.

## Supporting information

S1 Table Comparison of cutting resistance. (XLSX)

S2 Table The linear regression for the estimated against measured. (XLSX)
